# Multilevel Factors Associated with Social Isolation Trajectory Among Older Chinese Immigrants

**DOI:** 10.1093/geroni/igaf122.1626

**Published:** 2025-12-31

**Authors:** Ke Li, Fengyan Tang, BoRin Kim, Wendi Da, Yanping Jiang

**Affiliations:** Ohio University, Athens, Ohio, United States; University of Pittsburgh, Pittsburgh, Pennsylvania, United States; University of New Hampshire, University of New Hampshire, New Hampshire, United States; Rutgers University, New Brunswick, New Jersey, United States; Rutgers, The State University of New Jersey, New Brunswick, New Jersey, United States

## Abstract

Social isolation is particularly prevalent among older immigrants due to language and cultural barriers. However, the factors contributing to social isolation among this population remain understudied. This study aims to identify the patterns of social isolation change trajectory over eight years and to examine the factors at multiple ecological systems that are relevant to social isolation among older Chinese immigrants. Data came from five waves of the Population Study of Chinese Elderly in Chicago, with a study sample of 2,835 Chinese immigrants aged 60 years and older. Social isolation was measured by the social disconnectedness index composed of social network size and range, household size, living arrangement, and marital status. The examined multilevel factors included socio-demographic and health conditions, interpersonal relationships, immigration, and neighborhood characteristics. Latent class growth analysis (LCGA) identified four distinct change trajectories of social isolation, including “Persistent Low”, “Persistent Medium”, “Persistent High”, and “Increasing” social isolation. The results of multinominal logistic regression models indicated the unequal contributions of different factors to social isolation. Among older Chinese immigrants, factors including age, sex, social support, social strain, years in the U.S., sense of community, social cohesion, and neighborhood physical disorder were significantly associated with different social isolation change trajectories. These findings inform the need for culturally sensitive services that consider the effects of multifaced factors when addressing the issue of social isolation among older immigrants.

